# A rare missense variant abrogates the signaling activity of tetherin/BST-2 without affecting its effect on virus release

**DOI:** 10.1186/1742-4690-10-85

**Published:** 2013-08-10

**Authors:** Daniel Sauter, Dominik Hotter, Susanne Engelhart, Fabian Giehler, Arnd Kieser, Christian Kubisch, Frank Kirchhoff

**Affiliations:** 1 Institute of Molecular Virology, Ulm University Medical Center, 89081 Ulm, Germany; 2 Research Unit Gene Vectors, Helmholtz Zentrum München, German Research Center for Environmental Health, 81377 München, Germany; 3 Institute of Human Genetics, Ulm University Medical Center, 89081 Ulm, Germany

## Abstract

**Background:**

Tetherin (or BST-2) is an antiviral host restriction factor that suppresses the release of HIV-1 and other enveloped viruses by tethering them to the cell surface. Recently, it has been demonstrated that tetherin also acts as an innate sensor of HIV-1 assembly that induces NF-κB-dependent proinflammatory responses. Furthermore, it has been reported that polymorphisms in the promoter and 3‘ untranslated region of the *bst2* gene may affect the clinical outcome of HIV-1 infection. However, non-synonymous polymorphisms in the *bst2* open reading frame have not yet been described or functionally characterized.

**Results:**

Mining of the Exome Variant Server database identified seven very rare naturally occurring missense variants of tetherin (Y8H, R19H, N49S, D103N, E117A, D129E and V146L) in human populations. Functional analyses showed that none of these sequence variants significantly affects the ability of tetherin to inhibit HIV-1 virion release or its sensitivity to antagonism by HIV-1 Vpu or SIVtan Env, although Y8H alters a potential YxY endocytic motif proposed to play a role in virion uptake. Thus, these variants do most likely not represent an evolutionary advantage in directly controlling HIV-1 replication or spread. Interestingly, however, the R19H variant selectively abrogated the signaling activity of tetherin.

**Conclusions:**

Restriction of HIV-1 virion release and immune sensing are two separable functions of human tetherin and the latter activity is severely impaired by a single amino acid variant (R19H) in the cytoplasmic part of tetherin.

## Background

Tetherin (BST-2, CD317, HM1.24) is an interferon-induced host restriction factor that inhibits the release of HIV, Ebola, Lassa, Herpes and other enveloped viruses from infected cells by tethering nascent virions to the plasma membrane [1-5]. Tetherin is a dimeric type II transmembrane protein with a size of 30–36 kDa [6]. It contains a cytoplasmic N-terminal region, a transmembrane domain, a glycosylated coiled-coil extracellular domain, and a C-terminal glycosylphosphatidylinositol (GPI) anchor [6]. The unusual topology of this restriction factor with both a transmembrane domain and a GPI anchor allows it to directly tether budding virions to host cells with one membrane anchor sticking in the virion and the other one remaining in the cellular membrane [7]. The coiled-coil domain of tetherin seems to provide conformational flexibility to allow this anchoring process [8].

Most simian immunodeficiency viruses (SIVs), including the direct precursors of HIV-1 infecting chimpanzees and gorillas, use their accessory Nef protein to antagonize tetherin of their respective host species [9-11]. Human tetherin, however, contains a five amino acid deletion in its cytoplasmic domain that evolved in hominids after their divergence from chimpanzees [12] and confers resistance to Nef [9-11]. The pandemic major (M) group of HIV-1 managed to switch from Nef to Vpu to counteract the human tetherin orthologue [10]. In contrast, with a single documented exception [13], the rare HIV-1 group N, O, and P strains have apparently thus far failed to evolve effective antagonists during adaptation to humans [10,13-15]. Thus, efficient tetherin antagonism may have been a prerequisite for the efficient spread of the AIDS pandemic [16]. A recent study suggests that the cytoplasmic deletion not only rendered human tetherin resistant to Nef but also enhanced its ability to act as an innate sensor of HIV-1 assembly that induces NF-κB-dependent proinflammatory responses [17].

Like other antiviral host restriction factors, such as TRIM5α (tripartite motif 5-α) proteins that induce untimely uncoating of the viral capsid and APOBEC3G (apolipoprotein B mRNA-editing enzyme, catalytic polypeptide-like 3G) that causes lethal hypermutation of the viral genome, *bst2* shows evidence of positive selection [18,19]. It has been reported that polymorphisms in the human *trim5*α, *APOBEC3G* and *bst2* genes are associated with the clinical course of HIV-1 infection supporting a relevant role of these restriction factors *in vivo*[20-25]. Previous investigations of the *bst2* gene focused on variations in the promoter or 3’ untranslated region that may affect the expression levels of this restriction factor [25]. Here, we characterized seven rare variants of the human *bst2* gene that change the amino acid sequence of this restriction factor (Y8H, R19H, N49S, D103N, E117A, D129E and V146L). We demonstrate that one of these missense variants, R19H, disrupts the signaling activity of human tetherin without impairing its ability to restrict HIV-1 release.

## Results

### Non-synonymous polymorphisms in the human bst2 gene

**Figure 1:**
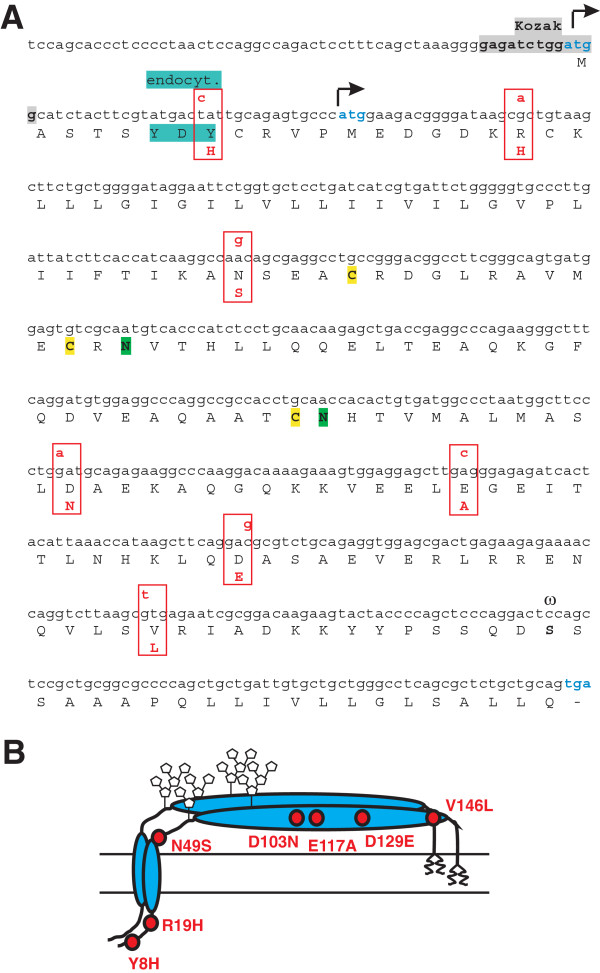
**Localization of missense variants in human tetherin. (A)** Codons containing non-synonymous variants in the tetherin gene are boxed in red. Non-synonymous exchanges are indicated above the nucleotide sequence and the predicted amino acid changes are shown below the protein frame. The Kozak sequence of the tetherin gene is highlighted in grey and both alternative start codons are indicated by arrows. Cysteine residues involved in dimerization and the two N-linked glycosylation sites are indicated in yellow and green respectively. ω marks the omega site of GPI anchor addition. **(B)** Schematic presentation of the position of the variants in regard to the structural elements of tetherin. Tetherin is depicted as a dimer and the two glycosylation sites are indicated.

A database search of the Exome Variant Server (http://evs.gs.washington.edu/EVS/), containing data on the exonic genetic variability of human genes as identified by exome sequencing of a several thousand individuals of European and African American descent, was performed in April 2012 to identify potential missense variants of tetherin. As expected from previous studies [18,19], we did not find any common non-synonymous polymorphisms in human *bst2* with a minor allele frequency (MAF) > 1%. However, the initial analysis allowed to identify eight very rare missense variants (MAF < 0.05%) in different human populations, although one of them (H68Y) was omitted from the database in subsequent releases and therefore probably was a false positive finding. Of the remaining seven missense variants, four (Y8H, R19H, N49S, D103N) were detected in people of European American descent, two (E117A, D129E) in African Americans and one (V146L) in both populations (Table 1). The single nucleotide polymorphisms (SNPs) are distributed throughout the *bst2* gene (Figure 1A). Two of the predicted amino acid changes (Y8H, R19H) are located in the cytoplasmic N-terminal region, one (N49S) adjacent to the transmembrane domain, and four (D103N, E117A, D129E and V146L) in the extracellular coiled-coil region (Figure 1B). Most of these alterations do not affect previously defined functional domains or structural motifs (Figure 1A), such as the GPI attachment signal, the two N-linked glycosylation sites or the three cysteine residues that are critical for homodimerization [6,26]. The exception is Y8H that affects a non-canonical YxY motif (Figure 1A). It has been reported that this motif is involved in the endocytic recycling of tetherin and that its ability to interact with adaptor protein complexes promotes virion uptake and subsequent degradation in lysosomes [27].

**Table 1:** Non-synonymous polymorphisms in the tetherin open reading frame

**Allele count**						
**Amino acid change**	**rsID**	**European American**	**African American**	**All**	**Conservation (phastCons)**	**Clinical link**
Y8H	rs141648094	G = 4/A = 8596	G = 0/A = 4406	G = 4/A = 13002	0.0	Not known
R19H	-	T = 2/C = 8598	T = 0/C = 4406	T = 2/C = 13004	0.0	Not known
N49S	rs144978205	C = 6/T = 8594	C = 0/T = 4406	C = 6/T = 13000	0.591	Not known
D103N	-	T = 1/C = 8599	T = 0/C = 4406	T = 1/C = 13005	0.0010	Not known
E117A	-	G = 0/T = 8600	G = 1/T = 4405	G = 1/T = 13005	0.135	Not known
D129E	-	C = 0/G = 8600	C = 2/G = 4404	C = 2/G = 13004	0.0	Not known
V146L	-	A = 3/C = 8597	A = 1/C = 4405	A = 4/C = 13002	0.0	Not known

#### Missense variants of human tetherin do not impair its ability to restrict virion release

**Figure 2:**
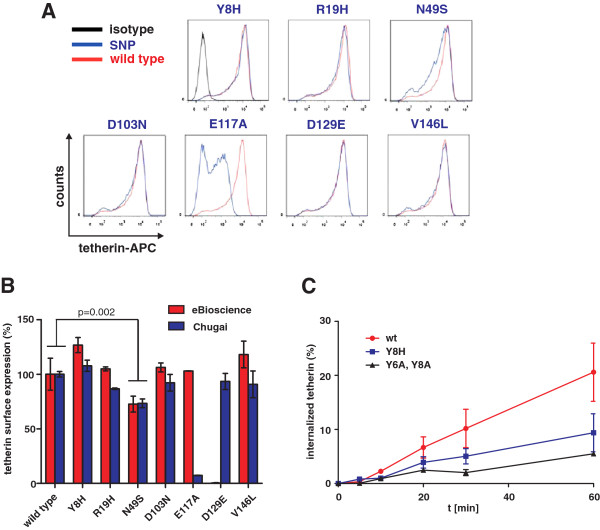
**Effect of variants on cell surface expression and internalization of tetherin. (A)** FACS analysis of 293T cells transfected with vectors expressing the indicated tetherin variants. Shown are results obtained with the antibody from Chugai Pharmaceuticals **(B)** Cell surface expression levels of all tetherin variants. Shown are average values ± SD (n = 2) of the mean fluorescence intensities (MFIs) measured for the indicated tetherin variants relative to those obtained for the wild-type tetherin protein (100%). **(C)** Internalization rate of surface tetherin in 293T cells transfected with the indicated tetherin variants. Shown are average values ± SEM (n = 2).

To examine whether these seven variants may affect the cell surface expression levels of tetherin, we transfected 293T cells with vectors expressing wild-type and mutant forms of tetherin and analyzed them by flow cytometry two days post transfection. Notably, we used two different antibodies for detection because the E117A substitution disrupted the previously described epitope (L116-L127) of the anti-BST-2 antibody from Chugai Pharmaceuticals [28] (Figure 2A) and the D129E substitution abrogated the interaction with the eBioscience antibody (Figure 2B). Our analyses showed that most tetherin variants were expressed as efficiently as wild-type tetherin on the cell surface (Figure 2B). Only the N49S substitution which is located just outside of the transmembrane domain (Figure 1B) significantly reduced the cell surface expression levels of tetherin. Western blot analysis confirmed that only the N49S variant significantly reduced the total levels of tetherin expression (Additional file 1: Figure S1). As expected, the D129E mutation disrupted the epitope of the eBioscience antibody and the E117A mutation prevented detection by the Chugai antibody. Although it has been reported that certain mutations in the cytoplasmic tail may affect the glycosylation pattern and maturation of tetherin [17] this was obviously not the case for these naturally occurring polymorphisms (Additional file 1: Figure S1A, B).

Since Y8H alters a previously described non-canonical YxY endocytosis signal [27], we also analyzed whether this mutation may affect the internalization rate of tetherin. We found that the Y8H variant showed a markedly reduced internalization rate compared to the major form of human tetherin (Figure 2C). However, the disruptive effect of the Y8H change alone was less severe than the combined mutation of both tyrosine residues (Y6A, Y8A).

**Figure 3:**
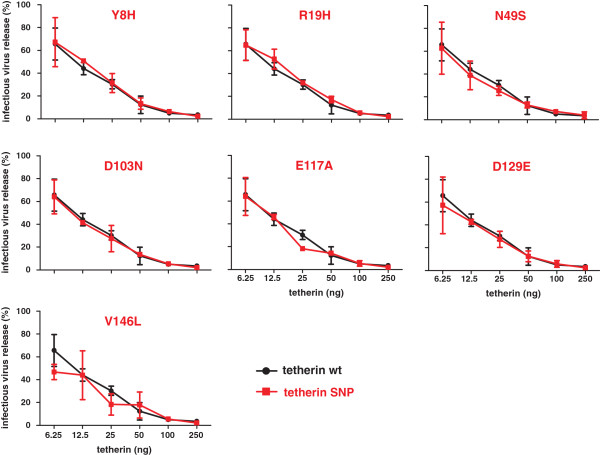
**Effect of variants on the ability of tetherin to restrict virion release.** Virus release from 293T cells following transfection with a *vpu*-deficient proviral HIV-1 NL4-3 construct and varying amounts of plasmids expressing the indicated tetherin variants. Infectious virus yield was determined by infection of TZM-bl indicator cells and is shown as a percentage of that detected in the absence of tetherin (100%). Shown are average values ± SD derived from two independent experiments, each performed in triplicate.

To test whether the amino acid variations affect the anti-retroviral activity of tetherin, we measured infectious virus yields from 293T cells following cotransfection of a *vpu*-deleted HIV-1 proviral construct [29] with various quantities of tetherin expression plasmid. We found that all tetherin variants potently inhibited infectious virus release in a dose-dependent manner and that even the N49S variant that showed modestly reduced levels of cell surface expression inhibited virion release as efficiently as wild-type tetherin (Figure 3). Thus, the seven rare missense variants analyzed had no significant effect on the ability of tetherin to restrict HIV-1 release.

#### Missense variants do not confer resistance to HIV-1 M Vpu or SIVtan Env

**Figure 4:**
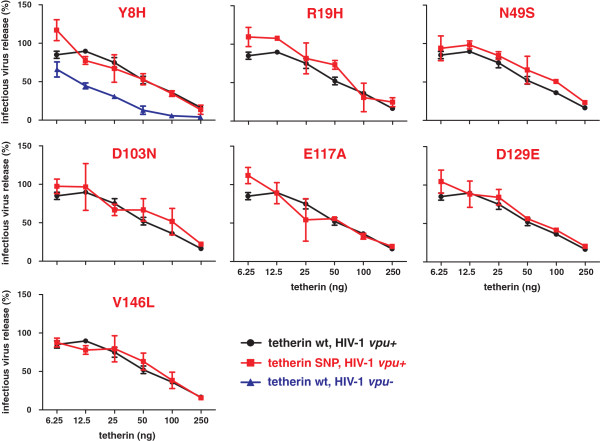
**Effect of variants on the susceptibility of tetherin to antagonism by Vpu.** Virus release from 293T cells following transfection with the wild-type proviral HIV-1 NL4-3 construct and varying amounts of tetherin expression plasmids. Results obtained with the *vpu*-deficient proviral construct are shown in the upper panel for comparison. Infectious virus release was determined as described in the legend of Figure 3. Shown are average values ± SD derived from two independent experiments, each performed in triplicate.

**Figure 5:**
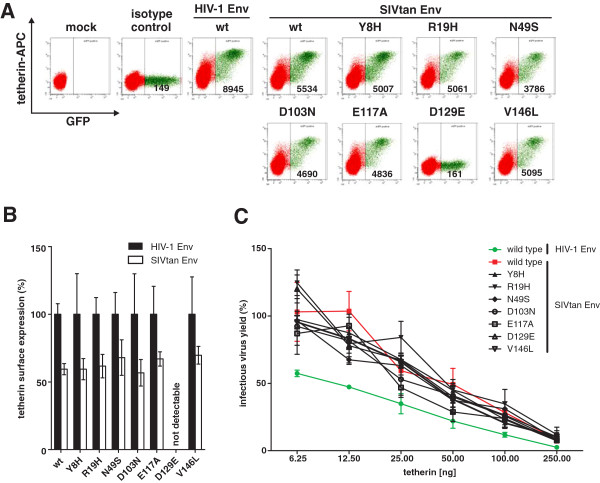
**Effect of variants on the susceptibility of tetherin to antagonism by SIVtan Env. (A)** FACS analysis of 293T cells transfected with vectors expressing the indicated tetherin variants and SIVtan or HIV-1 M NL4-3 Env. Shown are results obtained with the antibody from eBioscience. **(B)** Cell surface expression levels of all tetherin variants. Shown are average values ± SD (n = 3) of the mean fluorescence intensities (MFIs) measured in the presence of SIVtan Env relative to those obtained in the presence of HIV-1 M NL4-3 Env which served as negative control (100%). **(C)** Virus release from 293T cells following transfection with the *vpu*-deficient proviral HIV-1 NL4-3 construct, an expression vector for HIV-1 M or SIVtan Env and varying amounts of tetherin expression plasmids. Infectious virus release was determined as described in the legend of Figure 3. Shown are average values ± SD derived from one experiment, performed in triplicate.

Next, we examined the susceptibility of the tetherin variants to antagonism by Vpu by cotransfecting 293T cells with an HIV-1 proviral construct containing an intact *vpu* gene and constructs expressing the various tetherin proteins. As expected from previous studies [10], the release of wild-type HIV-1 was substantially less inhibited by tetherin than the *vpu*-defective derivative (Figure 4, upper panel). The seven tetherin variants were all as active against *vpu*-expressing HIV-1 as wild-type tetherin (Figure 4). This result implies that these missense variants do not affect the susceptibility of tetherin to counteraction by Vpu. Some primate lentiviruses use their Envelope (Env) proteins instead of Vpu to counteract tetherin by targeting the extracellular domain of tetherin [30,31]. Since most of the tetherin polymorphisms are located in the extracellular part we wondered whether they may confer resistance to Env. To test this, we analyzed the Envelope protein of SIVtan, infecting Tantalus monkeys, which has been shown to efficiently counteract human tetherin [30]. Experiments in transiently transfected 293T cells demonstrated that the SIVtan Env reduced the surface expression of all tetherin variants by about 40% (Figure 5A, B) and generally enhanced infectious virus release (Figure 5C). Thus, none of the polymorphisms affected the sensitivity of tetherin to SIVtan Env.

#### The R19H variant disrupts the signaling activity of human tetherin

**Figure 6:**
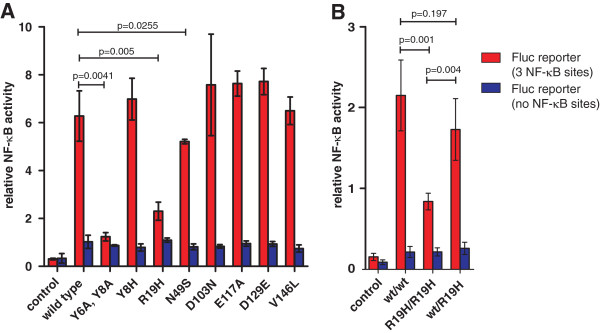
**Impact of variants on the ability of tetherin to activate NF-κB. (A)** Activation of NF-κB-dependent firefly luciferase reporter gene expression in 293T cells transiently cotransfected with tetherin, NF-κB-dependent (three NF-κB binding sites) or -independent firefly luciferase constructs and a reporter plasmid expressing gaussia luciferase under the control of a minimal promoter. The results show mean values ± SD of three independent transfections. **(B)** Equal amounts of vectors expressing wild-type and R19H tetherin were transfected to mimic heterozygosity. Mean values ± SD of four independent experiments in triplicates are shown.

In the following experiments we analyzed the impact of the seven naturally occurring variants on the signaling activity of human tetherin [17,32,33]. To examine this, we cotransfected 293T cells with tetherin expression plasmids, NF-κB dependent or independent firefly luciferase constructs and a reporter plasmid expressing gaussia luciferase under the control of a minimal promoter. Dual luciferase assays were performed and the firefly luciferase signals were normalized to the corresponding gaussia luciferase signals to compensate for differences in transfection efficiencies. In agreement with published data [17,33], wild-type tetherin strongly induced NF-κB activation and substitution of the N-terminal Y residues (Y6A, Y8A) disrupted this effect (Figure 6A). In contrast, alterations in the extracellular coiled-coil region of tetherin (D103N, E117A, D129E and V146L) did not significantly affect the ability of tetherin to promote NF-κB-dependent gene expression. Notably, the Y8H polymorphism did not reduce the signaling activity of tetherin either (Figure 6A) indicating that one N-terminal tyrosine-residue is sufficient for this function. In agreement with the modest reduction of its expression at the cell surface (Figure 2), the N49S tetherin variant showed a weakly but significantly reduced activity in NF-κB activation (Figure 6A). Most notably however, the R19H substitution significantly reduced the signaling activity of tetherin (Figure 6A). Thus, this positively charged arginine residue is critical for the ability of human tetherin to efficiently induce NF-κB-dependent gene expression but is dispensable for the restriction of HIV-1 virion release. We confirmed these findings using a reporter vector that contains six instead of three NF-κB binding sites and by using 293 instead of 293T cells (Additional file 2: Figure S2). To exclude artifacts due to high expression levels of tetherin, we titrated the tetherin expression vectors over two orders of magnitude. Activation of NF-κB by the R19H and Y6A, Y8A mutants was generally reduced (Additional file 2: Figure S2).

To determine tetherin-dependent NF-κB activation in case of heterozygosity we cotransfected expression vectors for wild-type and R19H tetherin in equal amounts. Activation of NF-κB was significantly higher in mixed wt/R19H samples compared to R19H tetherin alone and comparable to wild-type tetherin (Figure 6B). Thus, wild-type tetherin is acting in a dominant fashion and may rescue signaling in wt/R19H heterozygous individuals.

## Discussion

In this study, we functionally characterized seven very rare sequence variants of human tetherin. Our results show that none of them significantly reduces the ability of tetherin to restrict HIV release or its sensitivity to antagonism by HIV-1 Vpu or SIVtan Env. However, although these variants are very rare and do most likely not directly affect the control of HIV-1 replication or transmission, they led to two interesting observations. First, mutation of Y8H that affected a previously described YxY motif in the cytoplasmic tail of tetherin [27] did not impair its antiviral or signaling activity. Second, we found that substitution of R19H disrupts the signaling activity of human tetherin without reducing its capability to inhibit virus release. Thus, these two key functions of human tetherin are genetically separable.

Our finding that the Y8H substitution that shows a minor allele frequency of about 0.05% in Caucasians and seems to be absent in African Americans (Table 1) did not significantly affect the antiviral activity of human tetherin is in agreement with previous findings, showing that the presence of one of these two tyrosine residues and not the conservation of an YxY-based motif is critical for endocytosis of tetherin and its signaling activity [17,27,33]. Interestingly, however, the Y8H substitution reduced the internalization rate of tetherin albeit not as drastically as the double Y6A/Y8A mutation. Thus, our data confirm, that efficient endocytosis of tetherin is not required for the activation of NF-κB [17]. Notably, however, the relevance of the two N-terminal tyrosine residues for the steady state surface levels and endocytosis of human tetherin is somewhat controversial. Rollason and coworkers reported that tetherin was efficiently internalized if Y6 or Y8 were mutated individually, whereas the double mutant Y6A/Y8A was not endocytosed [34]. In our experiments, however, the Y8H change clearly reduced the internalization rate of tetherin (Figure 2C). Notably, Rollason *et al.* mutated the tyrosine residues to alanine and the introduction of the aromatic histidine residue may explain the discrepancy. Masuyama *et al.* performed similar analyses and described a slightly increased surface expression of the single mutants compared to wild-type tetherin but did not observe any change of the internalization rate [27]. A third study reported that even the double-mutant Y6A/Y8A was robustly endocytosed indicating that the YxY motif is not required for the constitutive internalization of tetherin [35].

The disruptive effect of the R19H substitution (MAF of 0.015%) on the signaling activity of tetherin came as a surprise since the Neil group has previously demonstrated that amino acids 9 to 11 (CRV) are critical for this function [17]. In the previous study alanine substitutions at positions 17 to 21 (DKRCK) impaired tetherin-mediated signaling but also reduced its steady state expression levels thus precluding meaningful functional analyses [17]. Notably, Galão and coworkers introduced triple alanine scanning mutations in the cytoplasmic domain of tetherin. Thus, their data do not contradict our finding that the arginine at position 19 is critical for the ability of tetherin to mediate efficient immune signaling but not for its antiviral activity or steady-state surface expression levels. It is tempting to speculate that both, residues 9 to 11 (CRV) and R19 may be important for the signaling activity of human tetherin because they are flanking a putative TRAF6 binding motif. The consensus motif (PxExx[Ar/Ac]) is only present in human tetherin and the CRV9-11 and R19 residues are flanking this site (*CRV*PMEDGDK*R*). Structural analyses have shown that at least eight amino acid residues (xxPxExx[Ar/(Ac]) are involved in TRAF6 binding [36], which may explain why mutation of CRV/AAA disrupted this interaction [17]. Interestingly, this putative TRAF6 binding motif emerged due to a hominid-specific deletion of the DDIWK motif in tetherin. This deletion is generally absent in tetherin orthologues of non-human primate species which induce little if any NF-κB activation [17]. Potentially, this may explain why deletion of the DDIWK motif enhanced NF-κB activation by an ape tetherin orthologue and *vice versa* its reintroduction into human tetherin severely impaired this signaling activity [17]. In contradiction to an important functional role of this putative TRAF6 interaction motif it has been reported that mutations in this region (P12-D17) do not disrupt tetherin-mediated signaling [17]. Notably, a recent study by Tokarev and colleagues could not co-immunoprecipitate tetherin with TRAF6 [37] and we were also unable to detect an interaction between tetherin and TRAF6 although we employed a variety of experimental systems including co-immunoprecipitations, reporter protein complementation assays and *in vitro* binding assays using purified proteins (data not shown). Thus, further studies on the functional impact of R19H, the humanoid-specific deletion, and the potential TRAF6 binding site in tetherin seem highly warranted.

The missense variants examined in the present study did not significantly affect the susceptibility of tetherin to antagonism by HIV-1 group M Vpu or SIVtan Env. It is noteworthy, however, that other viruses have also evolved antagonists of tetherin targeting the domains affected by these polymorphisms. For example, human Kaposi's sarcoma-associated herpesvirus (KSHV) encodes the RING-CH E3 ubiquitin ligase K5 that targets K18 in the cytoplasmic tail of tetherin for ubiquitination and subsequent degradation [38]. Similar to SIVtan, HIV-2 also uses its envelope glycoprotein to counteract tetherin by targeting the extracellular domain of this restriction factor [30,31]. Notably, the HIV-2 Env is only active against endogenous tetherin [30]. Thus, we were unable to determine whether the polymorphisms affect the susceptibility of tetherin to HIV-2 Env counteraction. In support of a selective pressure some of the positions that are polymorphic in humans or adjacent residues show variations between different primate species. For example, changes of YxY to CxY or FxY are found in the tetherin ortholgues of Sykes’ monkeys, talapoins and new world monkeys, respectively. Furthermore, talapoin tetherin contains the R19H changes analyzed in the present study and African green monkeys, Patas monkeys and Francois’ leaf monkeys contain a cysteine instead of an arginine residue at this position [39]. Thus, it will be of interest to further examine the effect of these variants analyzed on the susceptibility of tetherin to various viral antagonists.

The N49S substitution that was detected in European (MAF of 0.05%) but not in African Americans significantly reduced the tetherin surface expression and signaling activity by about 20% to 30% (Figures 2 and 6). We did not observe a significant effect of N49S on the anti-HIV-1 activity of human tetherin although the mutant tended to be slightly less active than the wild-type form (Figure 4). Recently, it has been reported that a 19-base-pair insertion polymorphism in the promoter region of *bst2* may be associated with faster disease progression and reduced expression levels of this restriction factor [25]. Whether or not the reduced surface expression of the N49S tetherin variant has an impact on the clinical outcome of HIV-1 infection remains to be determined and this will not be an easy task, given the rarity of this variant.

## Conclusions

We show that seven very rare sequence variants (Y8H, R19H, N49S, D103N, E117A, D129E and V146L) do not significantly affect the potency of human tetherin in inhibiting the release of HIV-1 particles or its sensitivity to antagonism by HIV-1 Vpu or SIVtan Env. The R19H variant, however, selectively abrogated the ability of tetherin to induce NF-κB-dependent gene expression. Thus, inhibition of virus release and immune sensing are separable functions of human tetherin and the arginine residue at amino acid position 19 plays a critical role in the latter activity.

## Methods

### Expression vectors

*Bst2* was cloned into the CMV promoter-based pCGCG expression vector coexpressing DsRed2 as previously described [10]. Single nucleotide polymorphisms were inserted using splicing by overlap extension PCR. To ensure expression of both isoforms [33] the genuine Kozak sequence was used. The pCAGGS vector expressing SIVtan Env was kindly provided by Ravindra Gupta [30]. The NF-κB firefly luciferase reporter plasmids containing three or six NF-κB binding sites were kindly provided by Bernd Baumann.

A minimal promoter gaussia luciferase construct was purchased from Clontech (#631909) and used for normalization. It contains the TATA-like promoter (pTAL) region from the Herpes simplex virus thymidine kinase (HSV-TK) that is not responsive to NF-κB. The gaussia luciferase in the pTAL vector was replaced by firefly luciferase using NcoI/XbaI and the resulting construct was used as a negative control for the NF-κB reporter plasmid.

### Proviral constructs

Generation of the HIV-1 NL4-3-based proviral construct coexpressing eGFP via an IRES and the *vpu*-deficient mutant thereof has been described previously [29,40].

### Cell culture and transfections

TZM-bl, 293T and 293 cells were grown under standard conditions in Dulbecco’s Modified Eagle Medium (DMEM) supplemented with 10% fetal bovine serum, antibiotics and L-glutamine. 293T and 293 cells were transfected using the calcium-phosphate precipitation method.

### FACS

To determine the cell surface expression levels of tetherin, 293T cells were transfected with expression vectors for GFP (1 μg) and tetherin (4 μg). To analyze the effect on SIVtan Env on tetherin surface expression levels, 293T cells were cotransfected with expression vectors for GFP (1 μg), tetherin (1 μg) and SIVtan Env (4 μg). Two days post transfection cells were analyzed by flow cytometry essentially as described previously [13]. Briefly, cells were stained with an unconjugated anti-BST-2 antibody from Chugai Pharmaceuticals or eBioscience and an APC-conjugated secondary anti-mouse antibody (Invitrogen). Fluorescence of stained cells was detected by two-color flow cytometry and tetherin expression was determined as mean fluorescence intensity of tetherin-APC in GFP expressing cells. Notably, the previously described epitope (L116-L127) of the antibody from Chugai Pharmaceuticals [28] is disrupted by the E117A substitution and the D129E substitution impaired the interaction with the eBioscience antibody (Figure 2).

### Western blot

To monitor tetherin expression, 293T cells were transfected with expression vectors for tetherin (2.5 μg) and GFP (2.5 μg). Two days post transfection cells were harvested, lysed in M-PER buffer (Thermo Scientific) and cell lysates were separated in 4-12% Bis-Tris gels (Invitrogen). After gel electrophoresis proteins were transferred to PVDF membranes and probed with an anti-BST-2 antibody from Chugai Pharmaceuticals or eBioscience. For internal controls, blots were incubated with antibodies specific for GFP (290-50, abcam) and β-actin (8227-50, abcam). Subsequently, blots were probed with anti-mouse or anti-rabbit IRDye Odyssey antibodies and proteins detected using a LI-COR Odyssey scanner.

### Internalization assay

To determine the internalization rate of tetherin, 293T cells were cotransfected with GFP and tetherin expression vectors. 40 h post transfection, surface tetherin was stained as described above and cells were incubated for 0, 5, 10, 20, 30 or 60 min at 37°C to allow internalization of tetherin. After incubation, surface staining was removed with an acidic wash (pH 2.0) in one half of the samples. The other half remained untreated for normalization. The amount of stained tetherin was quantified by flow cytometry. The internalization rate was determined by dividing the MFI of the internalized tetherin (samples with acidic wash) by the MFI of the cells without acidic wash.

### Virus release assay

To determine the ability of tetherin to restrict the release of infectious virions, 293T cells were seeded in six-well plates and transfected with 5 μg of HIV-1 NL4-3 IRES eGFP (wild-type or *vpu*-deficient) and different dilutions of tetherin expression vectors (250, 100, 50, 25, 12.5, and 6.25 ng). To determine the ability of SIVtan Env to antagonize tetherin, 293T cells were cotransfected with 4 μg of *vpu*-deficient HIV-1 NL4-3 IRES eGFP, 1 μg of vectors expressing SIVtan or HIV-1 M NL4-3 Env and varying amounts of tetherin expression vectors. Two days post transfection supernatants were harvested and the yield of infectious HIV-1 was determined by a 96-well infection assay on TZM-bl indicator cells.

### NF-κB reporter assay

Transfections for luciferase assays were performed in 96-well plates and each transfection was performed in triplicates. Tetherin expression plasmids (75 ng) were transfected in 293T cells along with NF-κB-dependent or -independent firefly luciferase constructs (125 ng) and a pTAL promoter gaussia luciferase reporter plasmid (25 ng). 48 h post transfection, dual luciferase assays were performed. Firefly luciferase signals were normalized to the corresponding gaussia luciferase signals.

### Statistical analysis

All statistical calculations were performed with a two-tailed unpaired Students-t-test using Graph Pad Prism Version 5.0. P values <0.05 were considered significant.

### Variant database

Single nucleotide variants were accessed from the Exome Variant Server, NHLBI GO Exome Sequencing Project (ESP), Seattle, WA (URL: ) (April 2012).

## Competing interests

The authors declare that they have no competing interests.

## Authors’ contributions

DS, DH and SE performed most experiments. CK carried out the data mining for SNPs. FG and AK participated in analyzing the interaction of tetherin with signaling molecules. DS and DH designed the study and performed the statistical analysis. FK and DS wrote the manuscript. All authors read and approved the final manuscript.

## Supplementary Material

Additional file 1 Figure S1 Expression of tetherin variants: 293T cells were transiently transfected with expression vectors for the indicated tetherin variants. Cells were lysed two days post transfection and total tetherin levels were determined by immunoblotting using anti-tetherin antibodies from (**A**) Chugai Pharmaceuticals or (**B**) eBioscience. GFP and β-actin served as transfection and loading controls, respectively. (**C**) The tetherin signal intensities were quantified and normalized to β-actin. The mean ± SEM of three independent blots incubated with the anti-BST2 antibody from Chugai Pharmaceuticals is shown. Click here for file

Additional file 2 Figure S2 Impact of variants on the ability of tetherin to activate NF-κB. (**A**) Activation of NF-κB-dependent firefly luciferase reporter gene expression in 293T or 293 cells transiently cotransfected with tetherin, NF-κB-dependent or -independent firefly luciferase constructs and a reporter plasmid expressing gaussia luciferase under the control of a minimal promoter. 3x and 6x NF-κB indicates a reporter vector with three or six NF-κB binding sites, respectively. Mean values ± SD of three independent transfections are shown. (**B**) Titration of the tetherin expression vectors. 293T cells were cotransfected with tetherin, NF-κB-dependent (three NF-κB binding sites) or -independent firefly luciferase constructs and a reporter plasmid expressing gaussia luciferase under the control of a minimal promoter. The mean of three independent transfections is shown. Click here for file
