# Supplementary figures and images for: A rare missense variant abrogates the signaling activity of tetherin/BST-2 without affecting its effect on virus release
Source: Retrovirology. 2013 Aug 10;10:85. doi: 10.1186/1742-4690-10-85 (PMC3751106; doi:10.1186/1742-4690-10-85)

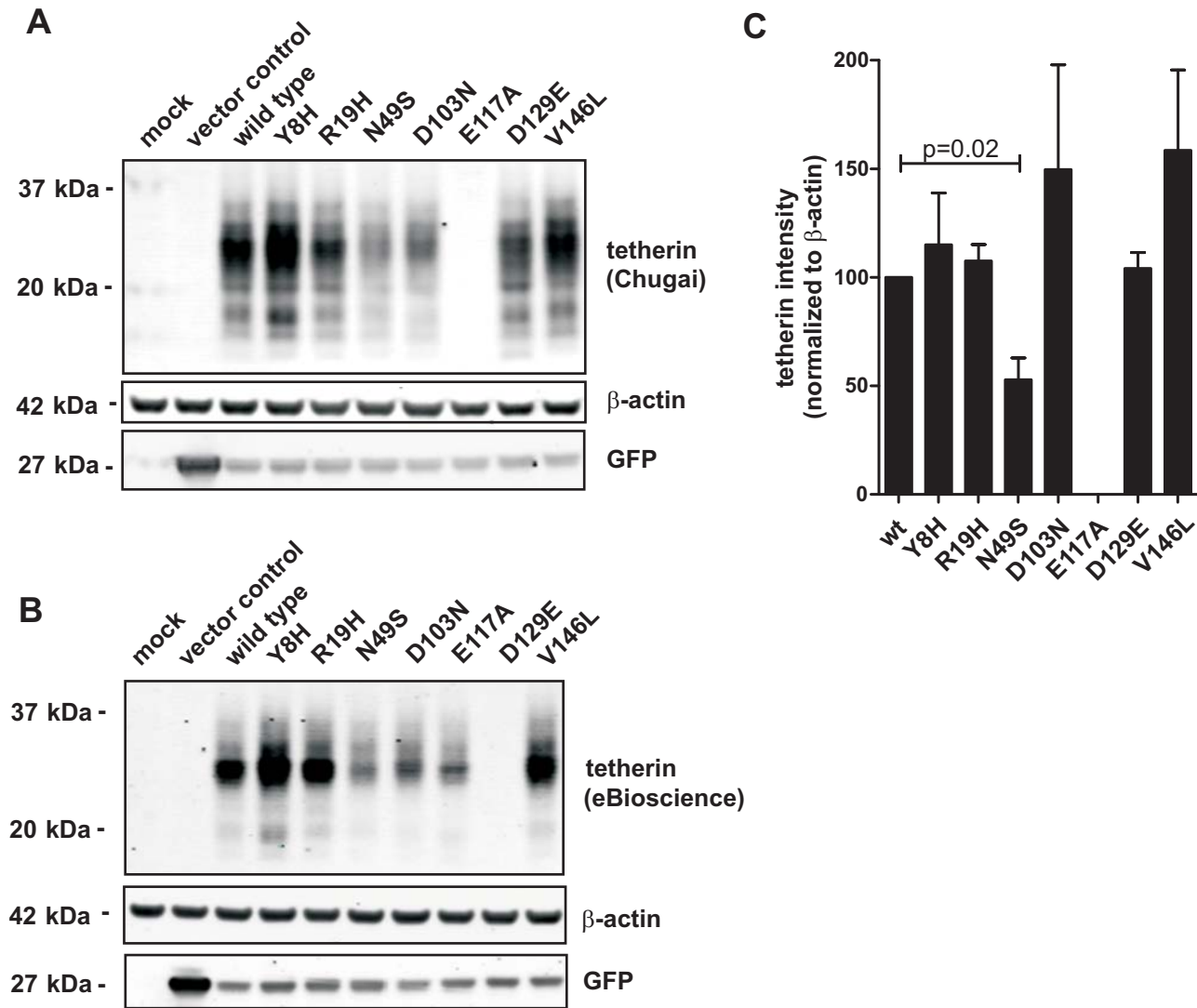

Supplement: Additional file 1 Figure S1 — Expression of tetherin variants: 293T cells were transiently transfected with expression vectors for the indicated tetherin variants. Cells were lysed two days post transfection and total tetherin levels were determined by immunoblotting using anti-tetherin antibodies from (A) Chugai Pharmaceuticals or (B) eBioscience. GFP and β-actin served as transfection and loading controls, respectively. (C) The tetherin signal intensities were quantified and normalized to β-actin. The mean ± SEM of three independent blots incubated with the anti-BST2 antibody from Chugai Pharmaceuticals is shown. [file 1742-4690-10-85-S1.pdf]

A

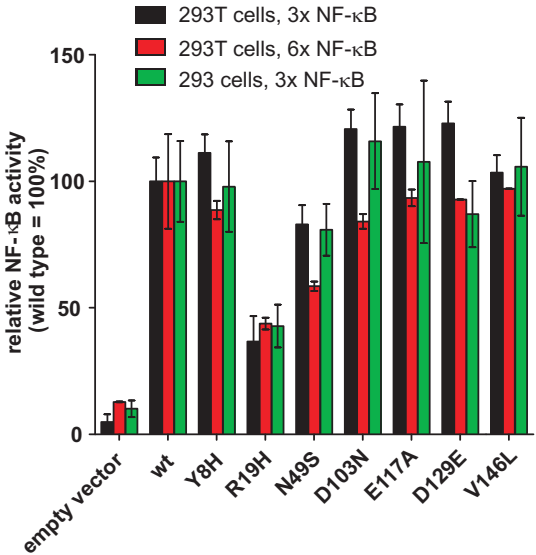

B

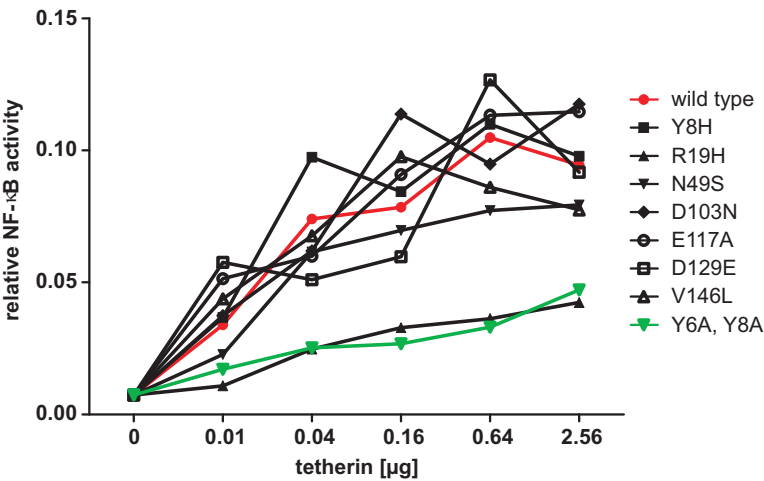

Supplement: Additional file 2 Figure S2 — Impact of variants on the ability of tetherin to activate NF-κB. (A) Activation of NF-κB-dependent firefly luciferase reporter gene expression in 293T or 293 cells transiently cotransfected with tetherin, NF-κB-dependent or -independent firefly luciferase constructs and a reporter plasmid expressing gaussia luciferase under the control of a minimal promoter. 3x and 6x NF-κB indicates a reporter vector with three or six NF-κB binding sites, respectively. Mean values ± SD of three independent transfections are shown. (B) Titration of the tetherin expression vectors. 293T cells were cotransfected with tetherin, NF-κB-dependent (three NF-κB binding sites) or -independent firefly luciferase constructs and a reporter plasmid expressing gaussia luciferase under the control of a minimal promoter. The mean of three independent transfections is shown. [file 1742-4690-10-85-S2.pdf]
